# Assessment of Methylene Chloride–Related Fatalities in the United States, 1980-2018

**DOI:** 10.1001/jamainternmed.2021.1063

**Published:** 2021-04-19

**Authors:** Anh Hoang, Kathleen Fagan, Dawn L. Cannon, Swati D. G. Rayasam, Robert Harrison, Dennis Shusterman, Veena Singla

**Affiliations:** 1 School of Medicine, University of California, San Francisco; 2 Office of Occupational Medicine and Nursing, Occupational Safety and Health Administration, Washington, DC; 3 Now Retired; 4 Program on Reproductive Health and the Environment, Department of Obstetrics, Gynecology and Reproductive Sciences, University of California, San Francisco; 5 Division of Occupational and Environmental Medicine, Department of Medicine, University of California, San Francisco; 6 Now with Healthy People and Thriving Communities, Natural Resources Defense Council, San Francisco, California

## Abstract

**Importance:**

Methylene chloride is a halogenated organic solvent widely used in paint strippers, cleaners, adhesives, and sealants. Despite label warnings and occupational standards, methylene chloride–related fatalities continue to occur in the United States.

**Objective:**

To identify and analyze methylene chloride–related fatalities in the US.

**Design, Setting, and Participants:**

For this case series, we conducted systematic searches of sources, including PubMed and government databases, for unintentional fatalities in the US that were associated with exposure to methylene chloride or products containing methylene chloride between 1980 and 2018. We reviewed all available information, including inspection reports, autopsy reports, and medical records; data analyses were conducted from August 2018 to August 2020. Cases were categorized as those occurring in the home (consumer deaths) or at work (occupational deaths).

**Exposures:**

Methylene chloride or products containing methylene chloride.

**Main Outcomes and Measures:**

To determine characteristics of the methylene chloride–related fatalities, we recorded demographic information; the setting; circumstances, including information on safety measures used, if available; and products used. Where medical records were available, we recorded toxicology results and autopsy findings. We also obtained data about nonfatal methylene chloride cases from the American Association of Poison Control Centers.

**Results:**

From 1980 to 2018, 85 methylene chloride–related fatalities were identified in the US, including 74 (87%) in occupational settings; of those who died, 75 (94%) were men, and for the 70 cases with available information, the median (interquartile range) age of the decedents was 31 (24-46) years. Paint strippers were the most common products involved in methylene chloride–related fatalities (n = 60). The proportion of occupational fatalities related to paint stripping increased from 22 (55%) before 2000 to 30 (88%) after 2000. Similarly, occupational fatalities associated with bathtub or paint stripping in bathrooms increased from 2 (5%) before 2000 to 21 (62%) after 2000. From 1985 to 2017, the American Association of Poison Control Centers documented 37 201 nonfatal methylene chloride cases, with a decrease in the annual number of cases starting in the late 1990s.

**Conclusions and Relevance:**

Results of this case series demonstrated that despite regulations to address the toxic effects of methylene chloride use for consumers and workers, there are continuing fatalities in the US, particularly in occupational settings. Prevention of fatalities associated with methylene chloride exposure should emphasize the use of safer substitutes, rather than hazard warnings or reliance on personal protective equipment.

## Key Points

**Question:**

What are the characteristics of fatalities associated with exposure to methylene chloride, a halogenated organic solvent widely used in paint strippers, cleaners, adhesives, and sealants in the United States?

**Findings:**

In a case series of 85 methylene chloride–related fatalities from 1980 to 2018, most deaths occurred at work. Although US regulatory policies have mandated product labeling and worker protections, fatalities continue to occur, with a greater proportion of recent deaths related to the use of paint-stripping products.

**Meaning:**

Prevention of methylene chloride–related fatalities should emphasize the use of safer substitutes, not hazard warnings or reliance on personal protective equipment.

## Teaser

This case series analyzes characteristics of methylene chloride–related fatalities in the United States from 1980 to 2018.

## Introduction

Methylene chloride (dichloromethane [CH_2_Cl_2_], Chemical Abstract Service Registry Number: 75-09-2) is a halogenated organic solvent widely used in paint strippers, cleaners, degreasers, adhesives, and sealants. The annual production volume in the United States is more than 200 million pounds.^1^ In the 1800s, methylene chloride’s narcotic effects were described related to anesthetic use; this use was discontinued owing to the narrow margin between doses leading to narcosis and death.^2,3^ In 1936, poisonings of US workers using methylene chloride–based paint strippers were reported.^4^ In 1952, a US fatality was reported in the peer-reviewed literature that involved a factory worker using methylene chloride as an extraction solvent.^3^ In 1976, Stewart and Hake^5^ described a patient hospitalized twice with myocardial infarctions following 2 separate uses of a consumer methylene chloride–based paint stripper. As neither the patient nor his physicians were aware of methylene chloride’s cardiotoxicity, the patient used the paint stripper a third time and died. The report emphasized the responsibility of physicians to inform patients of hazards of methylene chloride use in the absence of action by the US Environmental Protection Agency (EPA) or Consumer Product Safety Commission (CPSC).

The most serious acute effect of methylene chloride is narcosis, ranging from light-headedness, nausea, and headache to respiratory depression and death.^6,7,8,9,10,11,12,13,14,15^ Methylene chloride can also sensitize the myocardium to arrhythmias, a particular risk for people with cardiovascular disease.^5,16,17^ Other acute effects include skin burns from contact, corneal damage due to ocular splashes, and indirect consequences of narcosis (eg, falls or other trauma).^18^

Compared with other organic solvents, methylene chloride has a low boiling point (approximately 40 °C) with high equilibrium vapor pressure (349-440 mm Hg at 20-25 °C). Its vapors are heavier than air and accumulate in tanks, mixing vessels, and bathtubs.^19,20^ Methylene chloride is readily absorbed by ingestion, inhalation, and skin contact.^21,22,23,24,25^ After a time lag, hepatic metabolism of methylene chloride generates substantial quantities of carbon monoxide (CO), a chemical asphyxiant, and formaldehyde, a known carcinogen.^22,25^ In turn, CO exerts additive cardiotoxicity and neurotoxicity with native methylene chloride through competitive displacement of oxygen from hemoglobin, forming carboxyhemoglobin (COHb). Methylene chloride exposure also poses chronic health risks, including cancer; liver, kidney, and reproductive toxic effects; and cognitive impairment.^26^

**Figure 1.**
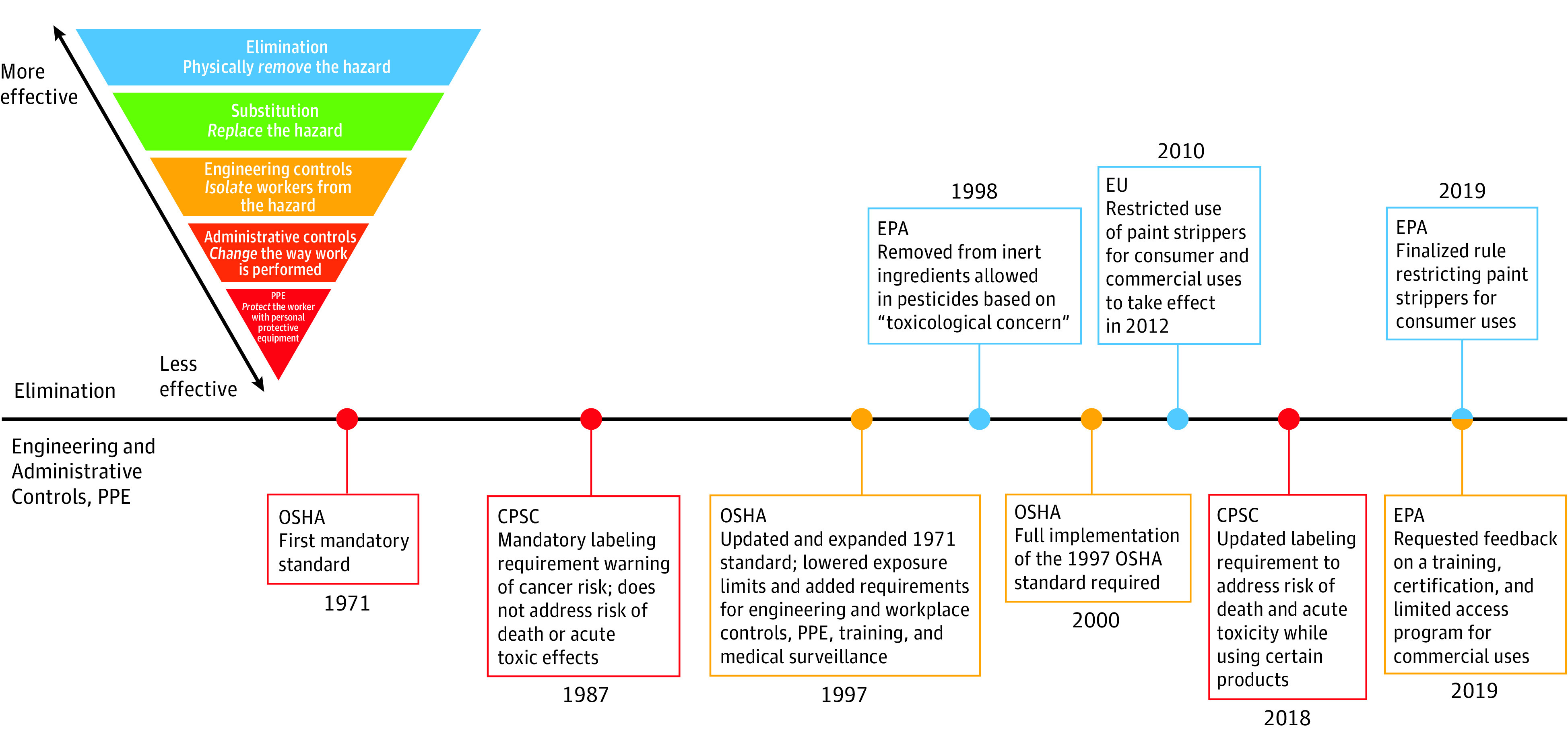
Timeline of Selected Policy Actions on Methylene Chloride Policies are categorized according to the National Institute for Occupational Safety and Health hierarchy of controls, with elimination being most effective in controlling hazards and personal protective equipment (PPE) being least effective.^27^ CPSC indicates the Consumer Product Safety Commission; EPA, US Environmental Protection Agency; EU, European Union; OSHA, Occupational Safety and Health Administration

In 1971, the Occupational Safety and Health Administration (OSHA), whose standards and regulations cover most private-sector employers, issued a permissible exposure limit (PEL) for methylene chloride that was intended to protect workers from acute narcotic and irritant effects (Figure 1^27^ and eTable 1 in the Supplement).^28^ In response to findings in the 1980s, the CPSC required labeling of methylene chloride products noting carcinogenicity (Figure 1 and eTable 1 in the Supplement).^29^ The labeling requirements did not cover the chemical’s acute effects, despite reports calling for such actions.^5,9^

To address carcinogenicity concerns, in 1997 OSHA revised its methylene chloride standard, lowering the PEL and requiring engineering and workplace controls, air monitoring, medical surveillance, and worker training. For exposures exceeding the PEL, OSHA required personal protective equipment (PPE), including full-face, pressure-demand, and supplied-air respirators (eTable 1 in the Supplement).^28,30^ In 2010, OSHA reviewed implementation of the 1997 standard and found that the standard “remains justified and necessary in light of ongoing hazards and fatalities.”^31^

In 2012, Chester et al^14^ reported a case series of 13 occupational methylene chloride–related deaths of bathtub refinishers occurring between 2000 and 2011. In all cases, the refinishers worked in poorly ventilated bathrooms, with inadequate or no PPE, and were found dead. The authors concluded that “safe use of a methylene chloride stripping agent in a small bathroom is unlikely.”^14^^(p121)^ The Box provides a case example of a typical fatality stemming from refinishing a bathtub with methylene chloride.^32,33,34,35^ In this case series, we identify and analyze methylene chloride–related fatalities in the US from 1980 to 2018 and also describe trends in nonfatal cases between 1985 and 2017.

Box. **Case Example**

In 2015, a 30-year-old man was refinishing a bathtub in a small, poorly ventilated bathroom in a public housing project with the bathroom door closed to keep vapors from escaping into the house.^32^ After 2 hours, the home leaseholder found him unconscious, slumped over the bathtub, and called 911. Resuscitation efforts were unsuccessful.

The worker used a paint stripper containing 85% to 90% methylene chloride. He was not wearing a respirator or other personal protective equipment. Results of the autopsy found acute liver, lung and kidney congestion, mild cerebral edema, moderate pulmonary edema, and cardiomegaly but no significant coronary atherosclerosis. Postmortem toxicology screening results were positive for several solvents, including a methylene chloride blood level of 89 µg/mL and a carboxyhemoglobin of 14%. No information on smoking history was available. Findings of a blood drug screen were negative except for tetrahydrocannabinol.

One month prior to his death, the worker had a syncopal episode while stripping another bathtub. His employer found and revived him, noting that the worker had slurred speech and burns (presumably chemical) on his face. After this episode, the employer reported providing the worker with a fan, a cloth scarf to protect against face and neck splashes, and a half-mask, powered air-purifying respirator for which the worker was expected to pay. When he died, neither a respirator nor a fan was found at the site or in the worker’s car.

The Occupational Safety and Health Administration (OSHA) was informed of the fatality and opened an inspection.^32^ On site the next day, testing found that methylene chloride levels both inside the bathtub (189 ppm) and in a bag containing rags the worker had used (370 ppm) exceeded OSHA’s short-term (15 minutes) exposure limit of 125 ppm. The employer was cited under several OSHA standards^33,34,35^ and fined $25 200.

## Methods

### Fatalities

#### Data Sources

The Committee for the Protection of Human Subjects of the University of California, San Francisco determined that this work did not meet the definition of human subjects research as defined by the Common Rule (45 CFR §46) and therefore did not require approval. We obtained data on methylene chloride–related fatalities for the period January 1, 1980, through December 31, 2018, from 10 different sources: PubMed, American Association of Poison Control Centers (AAPCC), OSHA, CPSC, LexisNexis, NewsBank, National Institute for Occupational Safety and Health (NIOSH) Fatality Assessment and Control Evaluation Program,^36^ the Social Security Death Index, and reports from the Center for Public Integrity^37^ and European Association for Safer Coatings Removal.^38^ More information on the search terms and the screening process are shown in eMethods and eFigure 1 in the Supplement.

#### Case Definition

We studied people in the US with acute exposure to a methylene chloride–containing product occurring between 1980 and 2018 as documented by inspection report, autopsy report, case report, environmental or biological measurement of methylene chloride, and/or a known metabolite of methylene chloride (CO, as indexed by COHb). There was no comparator. The outcome was unintentional death; suicides were excluded.

We defined occupational cases as those where the decedent was performing work for compensation and/or where exposure in a workplace either caused or contributed to the fatality, which included OSHA and non-OSHA cases. To define industry sectors, we used US Census Bureau data to cross-reference each industry sector to the most current system because the classification system for the industry codes has changed over time.^39^ Race and ethnicity data were used as demographic information because limited data were available.

#### Case Reconciliation

To determine whether each case was unique, we reconciled cases by matching the date of the incident with at least 1 other unique demographic identifier when available. Demographic identifiers included name, location, age, sex, and/or circumstance surrounding death.

#### Data Extraction and Analysis

Fatality incidents and details were recorded in Research Electronic Data Capture (REDCap) software (Vanderbilt University) with quality assurance and quality control performed by 1 author (S.D.G.R.) and 1 independent reviewer. Prism, version 9, software (GraphPad Software) was used for data synthesis and analyses. All hypothesis tests were 2-sided, with *P* < .05 considered significant. A χ^2^ test for trend was used to evaluate changes in fatality rates and their circumstances.

#### Pathology

Significant atherosclerotic coronary artery disease was defined as atherosclerotic stenosis of 50% or more in 1 or more coronary arteries. The included coronary arteries were left main, left anterior descending, left circumflex, or right coronary artery.

### Nonfatal Cases

We obtained data on methylene chloride incidents from the AAPCC (eFigure 1 in the Supplement). Cases of all ages were tabulated for nonintentional, nonfatal cases with health outcome coded as potential, minimal, minor, moderate, or major. Cases due to food poisoning, therapeutic errors, or unknown factor were excluded. Yearly numbers of cases were tabulated for total and occupational cases in Excel (Microsoft Corporation).

## Results

### Nonfatal Cases

**Figure 2.**
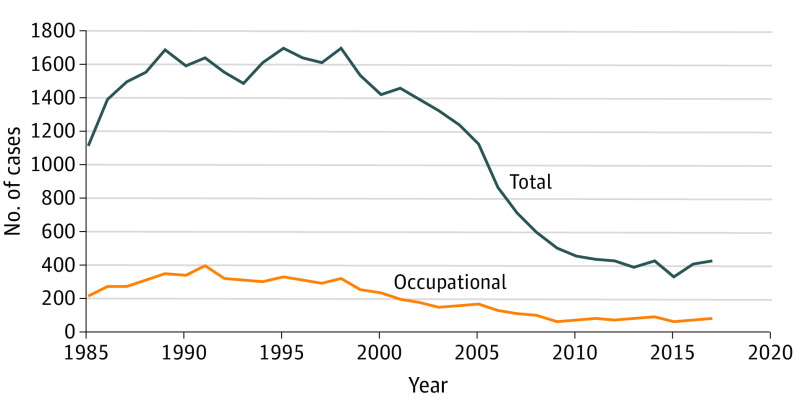
Methylene Chloride–Related Nonfatal Cases, 1985-2017 Data are from the American Association of Poison Control Centers.

From 1985 to 2017, the AAPCC documented 37 201 nonfatal methylene chloride cases, including 6589 occupational cases (Figure 2). The annual number of reported nonfatal cases peaked at 1701 cases in 1995. Subsequently, the annual number of cases decreased and reached a plateau level of about 408 cases a year between 2010 and 2017, including about 73 occupational cases.

### Fatal Cases

Between 1980 and 2018, we identified and analyzed 85 unique methylene chloride–related fatalities in the US (Table and eFigure 1 and eTable 2 in the Supplement). Of the fatalities, 75 (94%) were in men. For the 70 cases with available information, the median (interquartile range) age of the decedents was 31 (24-46) years (Table). Of the fatalities, 74 (87%) were occupational, of which OSHA investigated 55 (74%). In 5 cases, the worker had a prior on-the-job poisoning incident. There were 11 consumer fatalities. In the 78 cases with available information, the most commonly implicated products were paint strippers, cleaning and degreasing solvents, and adhesives or sealants (Table). In the 67 occupational cases with available information, 20 (30%) deaths occurred using equipment (eg, tanks, pits) found uniquely in the workplace. In 4 occupational incidents, there were multiple fatalities (range, 2-3 fatalities) (eTable 2 in the Supplement).

**Table.** Selected Characteristics of 85 Methylene Chloride–Related Fatalities Identified in the United States, 1980-2018^a^

Characteristic	No. (%)		
	Occupational fatalities (n = 74)	Consumer fatalities (n = 11)	Fatalities with autopsy reports available (n = 23)
Sex			
Total No. reported	69	11	23
Male	65 (94)	10 (91)	21 (91)
Female	4 (6)	1 (9)	2 (9)
Race/ethnicity			
Total No. reported	40	7	23
White	26 (65)	5 (72)	14 (61)
Hispanic	8 (20)	1 (14)	5 (22)
Black	6 (15)	1 (14)	4 (17)
Age			
Total No. reported	59	11	23
Median (IQR), y	31 (24-42)	45 (28-52)	37 (29-52)
Mean (SD), y	34 (13)	41 (18)	39 (13)
Range, y	18-64	14-80	20-62
Year of fatality			
Total No. reported	74	11	23
1980-1989	32 (43)	2 (18)	NA
1990-1999	8 (11)	3 (27)	NA
2000-2009	14 (19)	1 (9)	8 (35)
2010-2018	20 (27)	5 (46)	15 (65)
Product used			
Total No. reported	67	11	23
Paint stripper	52 (78)	8 (73)	23 (100)
Cleaning/degreasing solvent	12 (18)	1 (9)	NA
Adhesive/sealant	3 (4)	2 (18)	NA
Setting of incident			
Total No. reported	67	10	23
Bathroom	23 (34)	3 (30)	19 (82)
Industrial equipment	20 (30)	NA	2 (9)
Floor (nonbathroom)	4 (6)	1 (10)	2 (9)
Carpet	3 (4)	1 (10)	NA
Furniture	7 (11)	1 (10)	NA
Bystander on site^b^	3 (4)	NA	NA
Accidental ingestion	NA	1 (10)	NA
Other^c^	7 (11)	3 (30)	NA
Respirator use			
Total No. reported	36	4	21
No respirator used	20 (56)	2 (50)	13 (62)
Respirator used but not NIOSH approved	16 (44)	2 (50)	8 (38)

Abbreviations: IQR, interquartile range; NA, not applicable; NIOSH, National Institute for Occupational Safety and Health.

^a^ See eTable 2 in the Supplement for individual cases.

^b^ In 3 cases, the decedents did not use the products themselves but entered a room where vapors lingered after methylene chloride product use.

^c^ Other known fatality settings included working on cars, sheds, shutters, and trailers, or being at rest after product exposure.

Of the 85 total cases, 40 (47%) had information on PPE use. In the 36 occupational cases, a respirator was not used in 20 cases, and adequate respiratory protection (ie, a supplied-air respirator approved by NIOSH) was not used in 16 cases. Similarly, in 2 of the 4 consumer cases with information on PPE use, a respirator was not used, and in the other 2, the respirators were inadequate (Table). Information on whether decedents were trained on safe work practices for methylene chloride was not available.

**Figure 3.**
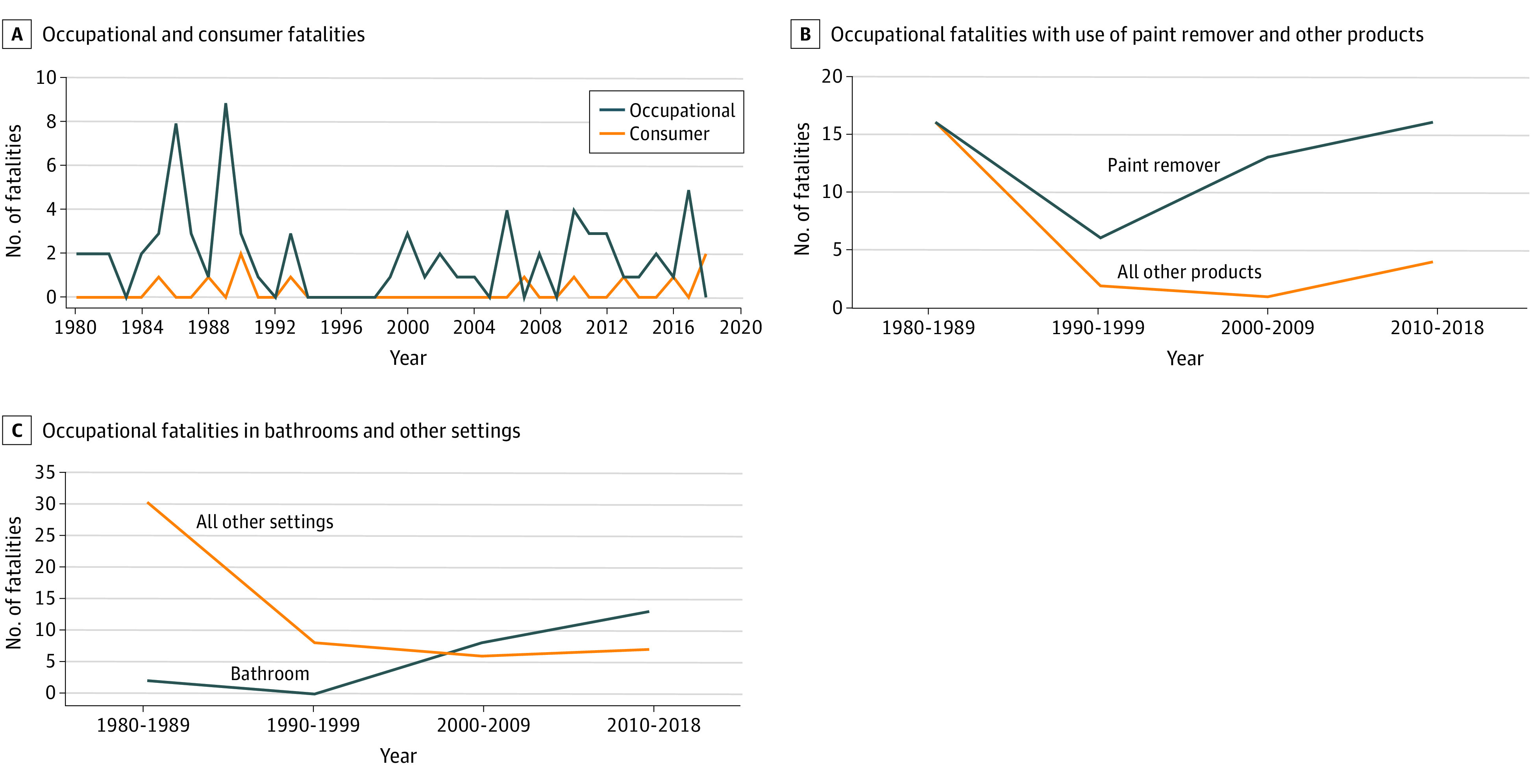
Methylene Chloride–Related Occupational and Consumer Fatalities, 1980-2018

There were no linear trends in the number of total fatalities, occupational fatalities, or consumer fatalities over the study period (χ^2^ test for trend, *P* = .21; Figure 3A and Table). Cases ranged from 0 to 9 per year, with no identified fatalities from 1994 through 1998. Full implementation of the 1997 OSHA standard was required by the year 2000 (Figure 1).^28^ Averaging by subperiod for all cases, from 1980 to 1999 we found a mean (SD) of 2.3 (2.6) cases per year (95% CI, 1.2-2.6 cases). From 2000 to 2018, the mean (SD) of cases was 2.1 (1.4) per year (95% CI, 1.4-2.8 cases).

We observed significant changes in the circumstances surrounding occupational deaths. The proportion of paint stripper–related deaths among workers increased compared with deaths due to other products (χ^2^ test for trend, *P* = .002; Figure 3B). Similarly, we found a significant increase in the proportion of fatalities occurring in bathrooms compared with other settings (χ^2^ test for trend, *P* < .001; Figure 3C). Consumer deaths followed a similar trajectory; bathroom and bathtub–related deaths increased from 0 of 5 before 2000 to 3 of 6 from 2000 to 2018.

Employer information was available for 56 (76%) of the occupational cases. Cases occurred in 7 industry sectors, as defined by the North American Industry Classification System (eTable 3 in the Supplement).^39^ Construction sector deaths increased from 5 cases (18%) before 2000 to 18 cases (64%) after 2000 (χ^2^ test for trend, *P* < .001; eTable 3 in the Supplement), with subindustry groups in construction all belonging to specialty trade contractors (eg, pouring concrete, site preparation, plumbing, painting, electrical work). Notably, 17 of the 18 cases after 2000 were bathroom related, and most of these involved bathtub refinishing. The geographic distribution of fatalities is shown in eFigure 2 in the Supplement.

### Pathology Results

Autopsy reports were available for 23 of the occupational fatalities (Table and eTable 4 in the Supplement). Mean and median body mass index values (calculated as weight in kilograms divided by height in meters squared) were in the overweight range (eTable 4 in the Supplement). Most livers were congested, with 9 (39%) having gross or microscopic changes consistent with hepatic steatosis. Of the steatotic livers, 7 were from an overweight (n = 1) or obese (n = 6) individual. Of the brains examined, 3 showed cerebral edema. Lungs showed congestion or edema.

The hearts of these deceased workers (n = 21) on average were 441 g heavier than reference values (233-383 g)^40^ but were within range when compared with an older population of men with cardiovascular disease (eTable 4 in the Supplement).^41^ Eleven (52%) workers had considerable atherosclerotic coronary artery disease. In 20 of the 23 autopsies, methylene chloride was detected in the blood, with results ranging from positive to 2200 µg/mL (eTable 5 in the Supplement). In 15 cases, blood COHb levels were measured, ranging from below the level of detection to 14% (eTable 5 in the Supplement).

## Discussion

In this case series, we found ongoing occupational and consumer fatalities with use of methylene chloride products in the US from 1980 to 2018, despite CPSC labeling requirements in 1987 and OSHA standards in 1997. Of the 85 deaths we documented, 74 were occupational. Fatalities typically occurred in bathroom settings and/or involved the use of paint strippers, particularly since 2000.

Although this review focused on methylene chloride–related fatalities, we obtained data on nonfatal cases from the AAPCC. Since the late 1990s, the annual number of reported cases trended downward before reaching a plateau level of about 408 cases a year between 2010 and 2017, including about 73 occupational cases. However, these data are limited because they are from a single source and only include acute nonfatal cases. Future studies should investigate the burden and trends of morbidities with methylene chloride (eg, lifelong disability, cancer risk).

Previous reports of methylene chloride–related fatalities generally have not examined pathology.^6,9,10,12,14^ Autopsies, although only available for 23 deaths in the present study, showed a greater proportion of cases with coronary artery disease (62%) than anticipated based on the published literature. For example, atherosclerotic coronary artery disease was found in 46% of autopsies in a military population older than 40 years.^42^ In a study of mortality incidence in 2538 patients (median age, 59 years; 70% men), 57% had atherosclerotic coronary artery disease determined by computerized tomography angiography, but only 15% had 50% or greater occlusive atherosclerotic coronary artery disease in any vessel^43^ as compared with 52% in the sample in the present study. The present results are consistent with previous findings that methylene chloride and its metabolic product (CO, as indexed by COHb) interact with preexisting cardiovascular disease to potentiate adverse health outcomes.^44,45,46,47^ The mechanism by which the direct neurotoxic effect of methylene chloride interacts with its cardiac effects and underlying heart disease merits further study and consideration in policy development.

Of the fatal occupational cases, 5 workers had prior on-the-job poisoning incidents with methylene chloride. These cases illustrate the challenges workers face in accessing the protections afforded them by law and the health care system. Clinicians should report occupational cases to OSHA and nonoccupational cases to the state health department. An occupational exposure history should be documented with an emphasis on current exposures. Although routine in occupational medicine practice, an occupational history should be part of general preventive care.^48,49,50^ At-risk patients should be counseled on the hazards of methylene chloride exposures and directed to resources on safer alternatives.^51,52,53^ Safer alternatives include benzyl alcohol, soy-based, and dibasic ester strippers.^51^ For secondary prevention, toxicological etiologies should be considered in the differential diagnosis of neurologic, cardiac, hepatic, respiratory, and dermatologic disorders. Clinicians should also ensure that workers with history of an acute solvent intoxication do not return to the same work situation without appropriate involvement from OSHA or other regulatory agencies.

Both OSHA and the CPSC require labeling of methylene chloride products. However, a 2016 review by the EPA found little scientific evidence to support the efficacy of labeling as a safety measure.^54^ The CPSC has also stated that “safety and warnings literature consistently identifies warnings as a less effective hazard-control measure than eliminating the hazard through design or guarding the consumer from a hazard.”^55^^(p59975)^ In 1981, Winek et al noted that “‘use with adequate ventilation’ is not descriptive enough to enable anyone to know what type of air exchange is required to prevent toxicity and lethality.”^9^^(p167)^ In the 40 occupational and consumer cases in the present study where information on respirator use was available, only 18 of the decedents used a respirator, and in each case the respirator was inadequate. Commonly available vapor cartridge respirators (eg, filtering facepiece or powered air-purifying respirator) do not provide sufficient protection against methylene chloride given the compound’s rapid saturation of filter elements.^30^ Case studies in occupational settings document permeation of methylene chloride through inadequate PPE.^7,56^

Despite requirements to fully implement the 1997 OSHA standard by 2000, results of this study found an increase in occupational fatalities associated with paint-stripping products after 1999. In 2010, OSHA’s assessment of compliance with the 1997 methylene chloride standard found that the number of firms with violations increased during the standard’s phase-in period.^31^ After 2000, this number of violations stabilized but did not decrease. The most common violations were failure to provide exposure monitoring, worker training on hazards and safe work practices, and appropriate PPE; OSHA noted that methylene chloride was most commonly used in paint removal but its usage had declined in other industries owing to substitution with other chemicals and/or technologies, likely driven by OSHA and EPA requirements.^31^

Additionally, OSHA does not have authority to prohibit uses of substances or chemicals; the EPA has these authorities under the Toxic Substances Control Act. In 2017, the EPA found that methylene chloride paint strippers posed unreasonable risks and proposed (but never finalized) a rule to prohibit these products in consumer and most commercial/industrial uses.^57^ In 2019, the EPA issued a final rule prohibiting consumer sale of methylene chloride paint strippers by the end of 2019, but the rule did not address commercial/industrial uses.^58^ The present analysis indicates that the EPA’s consumer sale prohibition, if compliance is achieved, may be effective in reducing consumer deaths because most consumer fatalities have been related to paint strippers. However, consumers are still at risk from other methylene chloride products implicated in fatalities (eg, adhesives or sealants and cleaning or degreasing solvents), which are still available for purchase.

The EPA’s 2019 rule does not address industrial/commercial uses of methylene chloride, thus the potential for occupational fatalities remains a major concern. The sector at greatest risk is the construction industry, particularly workers in bathrooms. For commercial uses, the EPA stated in 2019 that it was reevaluating options, including training, certification, and a limited access program (Figure 1 and eTable 1 in the Supplement).^59^

### Limitations

Methylene chloride–related fatalities are likely undercounted in the US owing to the fragmented nature of the public health reporting system. Because there is no unified data repository, we used heterogeneous sources to identify cases (eg, peer reviewed, government, gray literature), with each source having varying degrees of internal quality control and reporting standards (eMethods in the Supplement). Occupational fatalities may be underreported because not all workers are covered by OSHA. Consumer fatalities are likely underreported because there is no reporting requirement; the cases we discovered had media coverage. Thus, the relatively small number of cases may not reflect the actual trends over time. For example, we were unable to identify reasons for the 5-year gap in fatalities from 1994 to 1998. To identify existing and emerging environmental exposure risks, a unified reporting system should be developed to aggregate data from emergency departments, federal and state agencies, poison centers, and other relevant sources.^60^

Fatalities could be undercounted because fatal cardiovascular events subsequent to methylene chloride exposure may not be recognized as related to the chemical.^5^ Analysis of toxicology data from the 23 cases with autopsy information was limited by the heterogeneity of laboratory values from coroners’ offices (eTable 5 in the Supplement). In the forensic documentation of fatalities potentially attributable to methylene chloride, medical examiners should order toxicology tests for both methylene chloride and COHb.

## Conclusions

Due to the difficulty of mitigating acute risks, in 2012 the European Union prohibited methylene chloride–containing paint strippers for consumer and most commercial uses (Figure 1 and eTable 1 in the Supplement).^61^ The EPA’s 2020 evaluation of methylene chloride found that all consumer uses and most commercial uses variously exceeded health benchmarks of concern for acute, chronic, and cancer risks. The Toxic Substances Control Act requires that the EPA take action to effectively mitigate these risks (eTable 1 in the Supplement).^62^ According to the NIOSH hierarchy of controls (Figure 1), elimination is the most effective option to remove the hazards of methylene chloride. Occupational methylene chloride–related poisonings and deaths are preventable. Results of this case series indicate that a policy approach focused on hazard elimination and safer substitutes in consumer and occupational usages would be more effective in addressing fatalities than the current reliance on hazard communication and PPE.

## Notes

Supplement. eMethods. Search strategy for methylene chloride fatalities eTable 1. Timeline of major policy actions on methylene chloride in the US and EU eTable 2. List of included cases from search strategy for methylene chloride eTable 3. Occupational cases by industry sector eTable 4. Autopsy findings eTable 5. Available toxicology data from autopsies eFigure 1. Flowchart of the search and screening process for relevant methylene chloride fatality cases eFigure 2. Geographic distribution of methylene chloride fatalities in the U.S., 1980-2018
