# Supplementary material for: Assessment of Methylene Chloride–Related Fatalities in the United States, 1980-2018
Source: JAMA Intern Med. 2021 Apr 19;181(6):797–805. doi: 10.1001/jamainternmed.2021.1063 (PMC8056315; doi:10.1001/jamainternmed.2021.1063)
Supplement: Supplement. — eMethods. Search strategy for methylene chloride fatalities eTable 1. Timeline of major policy actions on methylene chloride in the US and EU eTable 2. List of included cases from search strategy for methylene chloride eTable 3. Occupational cases by industry sector eTable 4. Autopsy findings eTable 5. Available toxicology data from autopsies eFigure 1. Flowchart of the search and screening process for relevant methylene chloride fatality cases eFigure 2. Geographic distribution of methylene chloride fatalities in the U.S., 1980-2018 [file jamainternmed-e211063-s001.pdf]

Supplemental Online Content

Hoang A, Fagan K, Cannon DL, et al. Assessment of fatalities caused by methylene chloride exposure in the United States, 1980-2018. *JAMA Intern Med*. Published online April 19, 2021. doi:10.1001/jamainternmed.2021.1063

eMethods. Search strategy for methylene chloride fatalities
eTable 1. Timeline of major policy actions on methylene chloride in the US and EU
eTable 2. List of included cases from search strategy for methylene chloride
eTable 3. Occupational cases by industry sector
eTable 4. Autopsy findings
eTable 5. Available toxicology data from autopsies
eFigure 1. Flowchart of the search and screening process for relevant methylene chloride fatality cases
eFigure 2. Geographic distribution of methylene chloride fatalities in the U.S., 1980-2018

This supplemental material has been provided by the authors to give readers additional information about their work.

© 2021 Hoang A et al. *JAMA Intern Med*.

Table of Contents

eMethods. Search strategy for methylene chloride fatalities
eTable 1. Timeline of major policy actions on methylene chloride in the US and EU
eTable 2. List of included cases from search strategy for methylene chloride
eTable 3. Occupational cases by industry sector
eTable 4. Autopsy findings
eTable 5. Available toxicology data from autopsies
eFigure 1. Flowchart of the search and screening process for relevant methylene chloride fatality cases
eFigure 2. Geographic distribution of methylene chloride fatalities in the U.S., 1980-2018

© 2021 Hoang A et al. *JAMA Intern Med*.

eMethods. Search strategy for methylene chloride fatalities

The Center for Public Integrity, an investigative news organization, published a list of 56 fatal incidents involving methylene chloride with limited demographic and case information.[1] We used their work as an initial scaffold for building our case data. We searched through nine sources:

1. PubMed: PubMed is a free search engine accessing primarily the MEDLINE database of references and abstracts on life sciences and biomedical topics. The United States National Library of Medicine at the National Institutes of Health maintain the database as part of the Entrez system of information retrieval.[2]
2. American Association of Poison Control Centers (AAPCC): The American Association of Poison Control Centers is a national nonprofit organization representing each of the 55 poison control centers in the United States, the more than 1,700 professionals these centers employ, as well as other poison-related organizations.[3]
3. Occupational Safety and Health Administration (OSHA): OSHA is part of the United States Department of Labor and covers most private sector employers and their employees (self-employed and certain other categories of workers are excluded).[4]
4. Consumer Product Safety Commission (CPSC): The Consumer Product Safety Commission is an independent agency of the US government. CPSC operates the National Electronic Injury Surveillance System (NEISS) which collects surveillance data primarily from a probability sample of emergency departments.[5]
5. LexisNexis: Subscription service that includes access to legal briefs, motions, statutes, case judgements, opinions, law review and legal journal articles.[6]
6. NewsBank: NewsBank is a news database resource that provides archives of media publications as reference materials to libraries.[7]
7. Fatality Assessment and Control Evaluation (FACE) Program: This National Institute for Occupational Safety and Health (NIOSH) research program is designed to identify and study fatal occupational injuries by conducting surveillance and performing investigations, in collaboration with select participating states.[8]
8. European Association for Safer Coatings Removal: This industry association represents "the interests of producers of innovative products for safer coatings removal." The Association maintains a list of incidents related to methylene chloride products from selected countries including the US.[9]
9. Social Security Death Index: A database of death records created from the United States Social Security Administration's Death Master File Extract.[10]

We performed a systematic literature search in PubMed using the following search string: ("fatal outcome" OR "Cause of Death" OR "mortality" OR "fatality" OR "fatal" OR "death") AND ("Methylene Chloride/poisoning"[mh] or "methylene chloride/adverse effects"[mh] or "methylene chloride/toxicity"[mh] or dichloromethane). Our search string was not limited by publication date. If information such as the exact date of death and location was not in the published study, attempts were made to contact authors of all included studies to obtain the information.

© 2021 Hoang A et al. *JAMA Intern Med*.

We directly requested records related to fatal methylene chloride exposures from the AAPCC, OSHA, and CPSC. We also identified additional cases through OSHA's Integrated Management Information System (IMIS), a database with limited search functionality that provides investigation summaries. We used the following keywords: "methylene chloride" and "dichloromethane."

We consulted legal experts to help find additional case data by searching for those that resulted in litigation only available via access to the federal court docket. We conducted a systematic search for legal cases and news in LexisNexis with a simplified version of the search string used in PubMed: ("methylene chloride" or "dichloromethane") and "death." We used the same terms to look for media coverage of these fatalities in NewsBank. For both databases, we further limited our search to the date range 1/1/1980-12/31/2018 and U.S. sources. The FACE Program was manually searched for fatal exposures linked to methylene chloride. As the last step, we input the date of death, name, and age of the decedents already included in our study to cross-verify and fill in additional demographic data from the Social Security Death Index. Pathology data was analyzed for a subset of cases where autopsy information was available.

© 2021 Hoang A et al. *JAMA Intern Med*.

eTable 1. Timeline of major policy actions and authoritative body listings on methylene chloride in the US and EU

According to the National Institute for Occupational Safety and Health (NIOSH) hierarchy of controls, elimination is the most effective strategy, followed by engineering controls, then administrative controls, with Personal Protective Equipment (PPE) being the least effective. Regulatory actions result in mandatory requirements, while non-regulatory actions do not. Rows shaded in gray indicate regulatory proposals that have not yet been finalized.

Policy Action	Year Enacted	Hierarchy of Controls/ Authoritative Listing	Summary	Regulatory/ Non-regulatory
**U.S. Occupational Safety and Health Administration (OSHA) 29 CFR 1910.1000**	1971	Engineering/ Administrative Controls/PPE	The standard required employers to ensure that employee exposure does not exceed 500 ppm as an 8-hour time-weighted average (TWA), 1000 ppm as a ceiling concentration, and 2,000 ppm as a maximum peak for a period not to exceed 5 minutes in any 2 hours.	Regulatory; Mandatory for workers covered by OSHA.
**U.S. Consumer Product Safety Commission (CPSC) Statement of Policy for Methylene Chloride**	1987	Administrative Controls	"The purpose of the statement will be to notify manufacturers that provisions of the Federal Hazardous Substances Act require that their products be labeled to indicate that inhalation of methylene chloride vapor has produced cancer in certain laboratory animals and specify precautions to be taken during use by consumers."<br>CPSC provided guidance only and did not issue a rule with standard language for labeling.<br>This applies to any product containing methylene chloride (consumer, industrial, commercial). This labeling requirement makes no mention of risk of death via inhalation nor does it state the acute toxicity of inhaling methylene chloride vapors.	Regulatory; The labeling requirement is mandatory.
**NIOSH Immediately Dangerous to Life or Health (IDLH) Concentration**	1994	Engineering/ Administrative Controls	NIOSH revised the IDLH concentration to 2,300 ppm based on acute inhalation toxicity data in humans.	Non-regulatory

© 2021 Hoang A et al. *JAMA Intern Med*.

**U.S. OSHA 29 CFR Parts 1910, 1915, 1926**	1997	**Engineering/ Administrative Controls/PPE**	OSHA reduced the permissible exposure limit (PEL) to 25 ppm, 8-hour TWA and short-term exposure limit to 125 ppm (15-minute sampling). It stated that "the current PELs allow employee exposure to a significant risk of material impairment of health…The final rule also contains provisions for exposure control, personal protective equipment, employee exposure monitoring, training, medical surveillance, hazard communication, regulated areas, and recordkeeping. Together, these provisions will substantially reduce significant risk to the extent feasible."	Regulatory; Mandatory for workers covered by OSHA.
**U.S. EPA Inert Ingredients No Longer Used in Pesticides Products**	1998	**Elimination**	EPA removed methylene chloride from its list of pesticide product inert ingredients based on "toxicological concern."	Regulatory; Mandatory for any pesticide product registered in the U.S.
**EU Commission Regulation No 276**	2010	**Elimination**	"Paint strippers containing dichloromethane in a concentration equal to or greater than 0.1% by weight shall not be: placed on the market for the first time for supply to the general public or to professionals after 6 December 2010; placed on the market for supply to the general public or to professionals after 6 December 2011; used by professionals after 6 June 2012."	Regulatory; Mandatory for industrial, commercial and consumer paint stripper products.
**California Proposition 65**	2013	**Administrative Controls**	California found that methylene chloride is a carcinogen and requires warnings for products or uses exceeding the No Significant Risk Levels (NSRL) of 50 ug/day or 200 ug/day for inhalation exposure.	Mandatory in California for all products or uses that will result in exposures exceeding the NSRL.

© 2021 Hoang A et al. *JAMA Intern Med*.

**Massachusetts Toxic Use Reduction Act (TURA)**	2014	**Administrative Controls**	Methylene chloride was listed as a higher hazard substance (HHS) in 2014. The HHS designation lowers the threshold for reporting, planning, and paying fees under TURA to 1,000 pounds per year.	Regulatory. Requires Massachusetts companies that use large quantities of specific toxic chemicals to evaluate their operations, plan for pollution prevention, and report on the results each year.
**U.S. EPA Methylene Chloride and N-Methylpyrrolidone (NMP); Rulemaking under TSCA Section 6(a)**	2017	**Elimination**	Proposal to prohibit and restrict manufacture, processing, and distribution in commerce of methylene chloride for all consumer and most types of commercial paint and coating removal.	This regulation was never finalized, and was withdrawn Jan 15, 2021.
**U.S. CPSC Labeling of Certain Household Products Containing Methylene Chloride; Supplemental Guidance; Correction**	2018	**Administrative Controls**	Updating the 1987 CPSC guidance above, CPSC revised its statement to include acute hazards from inhalation in an enclosed space that lacks adequate ventilation in addition to the cancer risks.<br>However, CPSC again provided guidance only and did not issue a rule with standard language for labeling. There is no required text for labels. There are suggested text options that manufacturers can use if their products meet the labeling requirements.<br>This applies to any product containing methylene chloride (consumer, industrial, commercial).	Regulatory; The labeling requirement is mandatory.

© 2021 Hoang A et al. *JAMA Intern Med*.

**Retailer Phase-outs of Methylene Chloride-Containing Products**	2018	**Elimination**	Thirteen retailers, including Lowe's, Sherwin-Williams, The Home Depot, Amazon, and Walmart have agreed to stop selling paint strippers with methylene chloride in all stores globally by the end of 2018 (Lowe's, Sherwin Williams, The Home Depot, Amazon) or February 2019 (Walmart).<br>A 2019 investigation by Safer Chemicals Healthy Families found that Amazon, The Home Depot, and Walmart (among others) were still selling products both online and at retail locations. By the end of February 2019, The Home Depot was removing its remaining inventory of methylene chloride and reconfigured registers to block the sale of methylene chloride products. By March 2019, Amazon and Walmart had removed the non-compliant product pages.	Non-regulatory
**US EPA Methylene Chloride; Regulation of Paint and Coating Removal for Consumer Use Under TSCA Section 6(a)**	2019	**Elimination**	On March 27, 2019, EPA released a final rule determining that the use of methylene chloride in paint and coating removal poses an unreasonable risk of injury to consumer health. EPA also placed prohibitions and restrictions on the manufacture, processing, and distribution in commerce of methylene chloride for all consumer paint and coating removal.<br>This policy narrows the scope of the 2017 proposed rule regarding methylene chloride paint and coating removal products as it only applies to consumer products and does not include commercial uses.	Regulatory; Mandatory for paint and coating removal products for consumers.
US EPA Commercial Paint and Coating Removal Training, Certification and Limited Access Program: Methylene Chloride	2019	No specifics on the program provided yet	EPA released an Advance Notice of Proposed Rulemaking soliciting feedback on a Training, Certification, and Limited Access program to allow commercial use of methylene chloride.	This proposal is currently under review.
**US EPA Risk Evaluation for Methylene Chloride**	2020	EPA has not yet issued rule proposal(s)	EPA found unreasonable risks to consumers from all consumer uses of methylene chloride, and unreasonable risks to workers from most commercial uses of methylene chloride.	Non-regulatory; EPA is required by the Toxic Substances Control Act to take actions to mitigate risks identified

© 2021 Hoang A et al. *JAMA Intern Med*.

eTable 2: List of included cases from search strategy for methylene chloride

Dark and light gray shading indicates incidents where multiple fatalities occurred together.

Fatality	News-Bank	Pub-Med	EASCR	Center for Public Integrity	Legal Cases	OSHA	FACE	CPSC***	AAPCC***	Age	Sex	Setting of Death
1	No	No	Yes	Yes	No	Yes	No	No	No	20	M	Working at a finish-removal company
2	No	No	Yes	Yes	Yes	Yes	No	No	No	NA	M	Scraping varnish off the inside of a 500-gallon tank
3	No	Yes	No	No	No	No	No	No	No	20	M	Cleaning equipment in a tank
4	No	No	Yes	Yes	No	No	No	No	No	NA	NA	Working at a plastics and coatings company
5	No	No	Yes	Yes	No	No	No	No	No	NA	NA	Working at a coatings manufacturing company
6	No	No	No	No	Yes	No	No	No	No	26	M	Cleaning a press pit
7	No	No	Yes	Yes	No	No	No	No	No	NA	NA	Gluing carpet in a boat
8	No	No	Yes	Yes	No	Yes	No	No	No	NA	M	Cleaning a degreaser
9	No	No	No	No	No	No	No	No	Yes	14	M	Accidental ingestion of paint remover
10	No	No	No	No	No	No	No	No	Yes	38	M	Using a tile stripping agent on the floor
11	No	No	Yes	Yes	No	No	No	No	No	NA	NA	Cleaning a truck trailer
12	Yes	No	No	No	No	No	No	No	No	29	M	Using a spray gun under a house trailer
13	No	No	Yes	Yes	No	Yes	No	No	Yes	22	M	Stripping wooden furniture
14	Yes	No	Yes	Yes	No	Yes	No	No	No	23	M	Cleaning the inside of a tanker trailer

© 2021 Hoang A et al. *JAMA Intern Med*.

15	Yes	No	Yes	Yes	No	Yes	No	No	No	18	M	Cleaning the inside of a tanker trailer
16	Yes	No	Yes	Yes	No	Yes	No	No	No	32	M	Walking into a basement that had recent methylene chloride application
17	Yes	No	Yes	Yes	No	Yes	No	No	No	41	M	Checking on a coworker in the basement that had recent methylene chloride application
18	Yes	No	Yes	Yes	No	Yes	No	No	No	41	M	Stripping the basement floor
19	No	Yes	Yes	Yes	No	Yes	No	No	Yes	19	M	Stripping furniture
20	No	No	No	No	No	No	No	Yes	No	NA	M	Working at his paint stripping business
21	No	No	Yes	Yes	No	Yes	No	No	Yes	25	M	Checking the fluid level of a sump hole
22	No	No	Yes	Yes	No	Yes	No	No	No	NA	M	Cleaning a tank truck
23	No	No	Yes	Yes	No	Yes	No	No	No	NA	M	Replacing carpet in a boat.
24	No	No	No	No	No	No	No	No	Yes	48	M	Using a carburetor cleaner
25	No	No	No	No	No	No	No	No	Yes	30	NA	Using a carburetor and parts cleaner.
26	No	Yes	No	No	No	No	No	No	No	21	M	Stripping furniture in a dip tank
27	No	No	Yes	Yes	No	Yes	No	No	No	NA	M	Cleaning a tanker trailer with methylene chloride
28	No	Yes	Yes	Yes	No	Yes	No	No	No	29	M	Stripping a restroom floor
29	No	Yes	Yes	Yes	No	Yes	No	No	No	32	M	Stripping a restroom floor
30	No	No	Yes	Yes	No	Yes	No	No	No	NA	M	Working on top of a portable tank
31	No	No	Yes	Yes	Yes	Yes	No	No	No	31	F	Cleaning a 1600-gallon vat

© 2021 Hoang A et al. *JAMA Intern Med*.

32	No	No	Yes	Yes	No	Yes	No	No	No	34	M	Removing paint residue from a tote bin
33	No	No	Yes	Yes	No	Yes	No	No	No	21	M	Stripping away paint from metal registers
34	Yes	No	Yes	Yes	Yes	No	No	No	No	28	F	Stripping furniture in her attic
35	Yes	No	Yes	Yes	No	Yes	No	No	No	37	M	Removing paints and other finishes from furniture
36	Yes	No	Yes	Yes	No	Yes	No	No	No	36	M	Repairing a plastic-coated metal rack
37	No	No	No	No	No	Yes	No	No	No	NA	M	Found in the filter room of a resin mixing area with his face over a metal bucket that contained methylene chloride
38	No	No	Yes	Yes	No	Yes	No	No	No	NA	M	Stripping furniture
39	No	No	Yes	Yes	No	Yes	No	No	No	29	M	Stripping the floor of a squash court
40	No	No	Yes	Yes	No	Yes	No	No	No	21	M	Stripping the floor of a squash court
41	No	No	No	No	Yes	No	No	No	No	56	M	Installing carpet on his boat
42	No	No	Yes	Yes	No	Yes	No	No	No	NA	M	Cleaning a 250-gallon reactor vessel by pumping the solvent out of the tank
43	No	No	No	No	Yes*	No	No	No	No	24	M	Restoring his car in his garage
44	No	No	Yes	Yes	No	Yes	No	No	No	18	M	Stripping furniture
45	No	Yes	Yes	Yes	No	Yes	No	No	No	39	M	Stripping and re-glazing a bathtub
46	No	No	Yes	Yes	No	Yes	No	No	No	31	M	Stripping paint from shutters
47	No	Yes	Yes	Yes	No	Yes	No	No	No	29	M	Refinishing a bathtub

© 2021 Hoang A et al. *JAMA Intern Med*.

48	No	Yes	Yes	Yes	No	Yes	No	Yes	No	52	M	Stripping bathroom walls
49	No	No	No	No	No	No	No	Yes	No	64	M	Falling into a tank of paint remover at work
50	No	No	Yes	Yes	No	Yes	No	No	No	46	M	Stripping furniture
51	No	Yes	Yes	Yes	No	Yes	No	No	No	43	M	Stripping paint off of a porcelain tub
52	No	Yes	Yes	Yes	No	Yes	No	No	No	35	M	Refinishing a bathtub
53	No	Yes	Yes	Yes	No	Yes	No	No	No	57	M	Refinishing a bathtub
54	Yes	No	Yes	Yes	Yes*	Yes	No	No	No	28	M	Replacing carpet in the cabin of a boat
55	No	Yes	Yes	Yes	No	Yes	No	No	No	23	M	Stripping and re-glazing a bathtub
56	No	No	No	No	No	No	No	Yes	No	45	M	Stripping the walls in the bathroom
57	No	No	No	No	No	No	No	Yes	No	24	F	Cleaning inside a tank
58	No	Yes	Yes	Yes	No	Yes	No	No	No	27	F	Removing paint from a bathtub
59	No	No	No	No	No	No	No	No	Yes	31	M	Using a paint stripper
60	No	Yes	Yes	Yes	No	Yes	Yes	No	No	52	M	Refinishing a bathtub
61	No	Yes	Yes	Yes	No	Yes	Yes	No	No	24	M	Removing pool paint from a baptistery
62	No	Yes	Yes	Yes	No	Yes	No	No	No	31	M	Re-glazing a bathtub
63	No	Yes	Yes	Yes	No	Yes**	No	No	No	41	M	Using a paint stripper in a bathtub
64	Yes	Yes	Yes	Yes	No	Yes	No	No	No	49	M	Performing maintenance on a residential family dwelling
65	No	Yes	Yes	Yes	Yes*	Yes	No	No	No	30	M	Re-glazing a cast iron bathtub

© 2021 Hoang A et al. *JAMA Intern Med*.

66	Yes	Yes	Yes	Yes	Yes*	Yes	Yes	No	No	62	M	Scraping and removing old paint from the inside of the tank
67	No	No	Yes	Yes	Yes*	Yes	Yes	No	No	37	F	Removing the old coating from a bathtub
68	No	No	No	No	No	Yes	No	No	No	57	M	Exposure to methylene chloride at their workplace
69	Yes	No	Yes	Yes	No	Yes	No	No	No	37	M	Cleaning a tank
70	No	No	Yes	Yes	No	No	No	Yes	Yes	80	M	Using a paint remover in a shed
71	No	No	Yes	Yes	No	Yes	Yes	Yes	Yes	50	M	Performing a bathtub resurfacing that involved the removal of the second layer of bathtub coating.
72	No	No	Yes	Yes	No	Yes	No	No	No	20	M	Refinishing a bathtub.
73	No	No	No	No	No	No	No	No	Yes	56	M	Acute methylene chloride exposure.
74	Yes	No	Yes	Yes	No	Yes	No	No	No	30	M	Refinishing a bathtub
75	No	No	No	No	No	Yes**	No	No	No	60	M	Refinishing a bathtub
76	No	No	No	No	No	No	No	Yes	No	48	M	Sealing bathroom shower tiles
77	No	No	No	No	No	Yes	No	No	No	49	M	Stripping an aluminum bracket
78	Yes	No	No	No	No	Yes	No	No	No	21	M	Stripping a bathtub
79	No	No	No	No	No	Yes	Yes	No	No	43	M	Removing old glaze from a bathtub
80	Yes	No	No	No	No	Yes	No	No	No	31	M	Refinishing the floor
81	No	No	No	No	No	Yes	No	No	No	47	M	Cleaning a tank
82	Yes	No	No	No	No	No	No	No	No	31	M	Refinishing the front fork of his bike in a bathtub
83	No	No	No	No	No	No	No	No	No	52	M	Helping to restore a friend's property for sale

© 2021 Hoang A et al. *JAMA Intern Med*.

84	No	No	No	No	No	No	No	No	Yes	NA	M	Using a paint stripper
85	No	No	No	No	No	No	No	No	Yes	30	M	Using a paint stripper

*These legal cases were provided by the collaborating law firm and can be made available upon request.
**These OSHA cases were provided by collaborating partners at the Administration and can be made available upon request.
***These exposure cases were provided using a records request from the designated agency/ organization.

© 2021 Hoang A et al. *JAMA Intern Med*.

eTable 3: Occupational Cases by Industry Sector

	Number of Fatalities		
	1980-1999 (n=28)	2000-2018 (n=28)	Total (n=56)
Construction	5 (18%)	18 (64%)	23 (41%
Manufacturing	6 (21%)	6 (21%)	12 (22%)
Retail & Wholesale Trade	2 (7%)	1 (4%)	3 (5%)
Transportation and Warehousing	3 (11%)	0 (0%)	3 (5%)
Administrative and Support and Waste Management and Remediation Services	3 (11%)	0 (0%)	3 (5%)
Other Services (except Public Administration)	8 (29%)	3 (11%)	11 (20%)
Public Administration	1 (3%)	0 (0%)	1 (2%)

© 2021 Hoang A et al. *JAMA Intern Med*.

eTable 4. Autopsy findings [11, 12]

Overweight = body mass index 25 to 29.9 kilogram/square meter
Obese = body mass index over 30 kilogram/ square meter

(a) Organ weights

Organ	Mean weight in grams (SD)<br>Median weight in grams (IQR)	(Min-Max)	Reference Mean weight in grams (SD) and reference range		P-value
Brain (N=23)	1470 (140)<br>1430 (1380, 1540)	(1220-1750)	1407 (123.5)<br>------------	(1179-1621)	**0.048**
Heart (N=21*)	441 (94)<br>418 (375, 536)	(260-580)	331 (56.7)<br>------------	(233-383)	**<0.0001**
Lungs (N=22**)	Right Lung<br>681 (229)<br>650 (580, 825)	(330-1290)	445 (159)<br>------------	(155-720)	**<0.0001**
	Left Lung<br>583 (179)<br>570 (490, 690)	(250-850)	395 (147)<br>------------	(112-675)	**<0.0001**
Liver (N=23)	2244 (494)<br>2238 (1860, 2600)	(1490-3090)	1561 (317)<br>------------	(968-1860)	**<0.0001**

*two hearts not included due to organ donation before autopsy
**one pair of lungs not included due to organ donation before autopsy

© 2021 Hoang A et al. *JAMA Intern Med*.

(b) Heart Autopsy Findings Compared to Overweight Subgroup[13]

Organ	Mean weight in grams (SD)<br>Median weight in gram (IQR)	(Min-Max)	Reference Mean weight in grams (SD) and reference range		P-value
Heart (N=21)	441 (94)<br>418 (375, 536)	(260-580)	344 (43.8)	(N/A)	**<0.0001**

Two hearts had ASCAD of less than 50% stenosis in one or more vessels. Two hearts were unavailable for examination, and in one heart, the degree of atherosclerosis was undefined.

(c) Autopsy BMI Comparisons[14]

BMI	Mean weight in grams (SD)<br>Median weight in grams (IQR)	(Min-Max)	Reference Mean weight in grams (SD) and reference range		P-value
BMI (N=22)	27.9 (5.7)<br>27 (22.5, 33.3)	(20.6-36.8)	25.4 (4.9)	(18.5-24.9)	0.07

© 2021 Hoang A et al. *JAMA Intern Med*.

eTable 5. Available toxicology data from autopsies

All decedents used methylene chloride paint remover products. LOD= level of detection

Year	Age	Sex	% Carboxyhemoglobin (COHB) in blood	Methylene chloride level (mcg/mL)
2001	29	M	Not performed	Positive*
2002	52	M	<LOD	94
2004	43	M	Not performed	Not performed
2006	35	M	3	223
2006	57	M	<LOD	100
2006	23	M	<LOD	Positive*
2007	45	M	<5	Positive*
2008	27	F	Not performed	99
2010	52	M	<LOD	50
2010	24	M	10	378
2011	49	M	Not performed	18
2011	30	M	8	Negative
2011	62	M	<5	2200
2012	37	F	Not performed	120
2014	20	M	<5	Positive*
2015	30	M	14	89
2016	60	M	Not performed	78
2017	21	M	3	89
2017	43	M	10	Positive*
2017	31	M	<LOD	77

© 2021 Hoang A et al. *JAMA Intern Med*.

2017	49	M	<5	Not performed
2018	31	M	Not performed	190
2018	52	M	Not performed	Positive*

*quantitative testing not performed

[1] Hopkins JS. Common solvent keeps killing workers, consumers. Center for Public Integrity. https://publicintegrity.org/inequality-poverty-opportunity/workers-rights/common-solvent-keeps-killing-workers-consumers/. Published September 21, 2015. Accessed November 11, 2020.

[2] National Library of Medicine. PubMed. National Center for Biotechnology Information. https://pubmed.ncbi.nlm.nih.gov/about/. Accessed November 11, 2020.

[3] American Association of Poison Control Centers. About. https://aapcc.org/about. Accessed November 11, 2020.

[4] Occupational Safety and Health Administration. Workers Rights - OSHA 3021-06R. https://www.osha.gov/Publications/osha3021.pdf. Published 2017. Accessed November 11, 2020.

[5] Consumer Product Safety Commission. National Electronic Injury Surveillance System (NEISS). CPSC.gov. https://www.cpsc.gov/Research--Statistics/NEISS-Injury-Data. Published August 11, 2020. Accessed November 11, 2020.

[6] LexisNexis. https://www.lexisnexis.com/en-us/gateway.page. Accessed November 11, 2020.

[7] NewsBank. About NewsBank. https://www.newsbank.com/about-newsbank. Published September 18, 2020. Accessed November 11, 2020.

[8] National Institute for Occupational Safety and Health. Fatality Assessment and Control Evaluation (FACE) Program. Centers for Disease Control and Prevention. https://www.cdc.gov/niosh/face/default.html. Accessed November 11, 2020.

[9] European Association for Safer Coatings Removal. DCM incidents-Paint Stripping in the EU-DCM paint strippers. https://www.eascr.org/dcmincidents.html. Published 2004. Accessed November 11, 2020.

[10] Social Security. Data Exchange – Requesting SSA's Death Information. https://www.ssa.gov/dataexchange/request_dmf.html. Accessed November 11, 2020.

[11] Molina D, DiMaio V. Normal organ weights in men: part I-the heart. Am J Forensic Med Pathol 2012;33: 362-367.

[12] Molina D, DiMaio V. Normal organ weights in men: part II-the brain, lungs, liver, spleen, and kidneys. Am J Forensic Med Path 2012;33(4):368-372.

[13] Molina D, DiMaio V. Normal organ weights in men: part I-the heart. Am J Forensic Med Pathol 2012;33: 362-367.

[14] Molina D, DiMaio V. Normal organ weights in men: part I-the heart. Am J Forensic Med Pathol 2012;33: 362-367.

© 2021 Hoang A et al. *JAMA Intern Med*.

eFigure 1: Flowchart of the search and screening process for relevant methylene chloride fatality cases.

*Cases, articles and reports were reconciled as they may contain more than one fatality, or the same fatality may have been reported across databases.

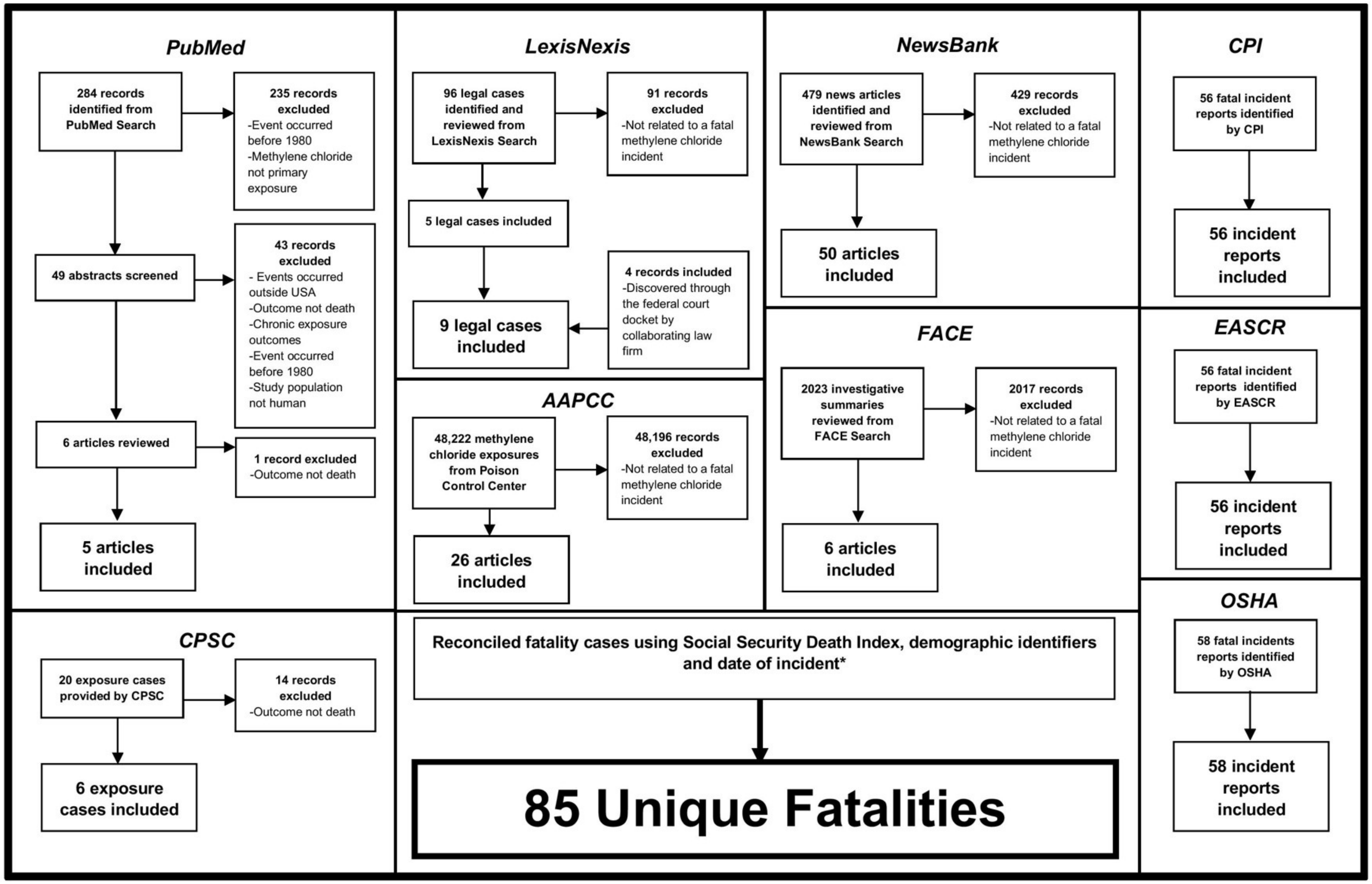

© 2021 Hoang A et al. *JAMA Intern Med*.

eFigure 2: Geographic distribution of methylene chloride fatalities in the US, 1980-2018

Red markers show fatalities with known locations. The majority of cases (66%) took place east of the Mississippi River. Illinois had seven fatalities located in Cook County. The second highest tally of fatalities were in Ohio and Pennsylvania respectively with 6 cases each.

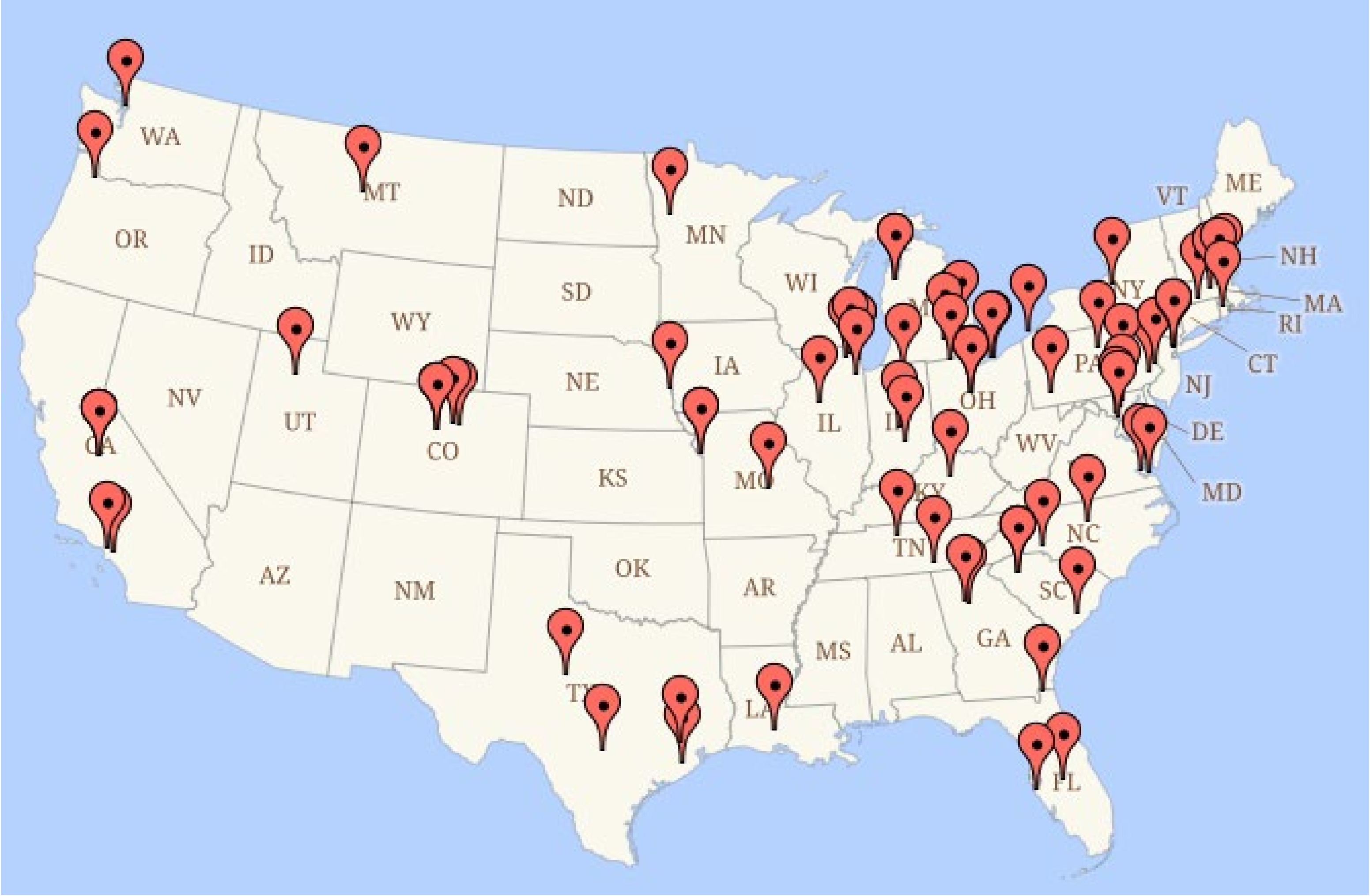

© 2021 Hoang A et al. *JAMA Intern Med*.
